# Effect of Low Refocusing Angle in T_1_-Weighted Spin Echo and Fast Spin Echo MRI on Low-Contrast Detectability: A Comparative Phantom Study at 1.5 and 3 Tesla

**DOI:** 10.1155/2013/680292

**Published:** 2013-08-06

**Authors:** Subhendra N. Sarkar, Jason L. Mangosing, Pooja R. Sarkar

**Affiliations:** ^1^Department of Radiology, Beth Israel Deaconess Medical Center, Harvard Medical School, 330 Brookline Avenue, Boston, MA 02215, USA; ^2^School of Medicine, University of Texas Health Science Center, 7703 Floyd Curl Drive, San Antonio, TX 78229, USA

## Abstract

MRI tissue contrast is not well preserved at high field. In this work, we used a phantom with known, intrinsic contrast (3.6%) for model tissue pairs to test the effects of low angle refocusing pulses and magnetization transfer from adjacent slices on intrinsic contrast at 1.5 and 3 Tesla. Only T_1_-weighted spin echo sequences were tested since for such sequences the contrast loss, tissue heating, and image quality degradation at high fields seem to present significant diagnostic and quality issues. We hypothesized that the sources of contrast loss could be attributed to low refocusing angles that do not fulfill the Hahn spin echo conditions or to magnetization transfer effects from adjacent slices in multislice imaging. At 1.5 T the measured contrast was 3.6% for 180° refocusing pulses and 2% for 120° pulses, while at 3 T, it was 4% for 180° and only 1% for 120° refocusing pulses. There was no significant difference between single slice and multislice imaging suggesting little or no role played by magnetization transfer in the phantom chosen. Hence, one may conclude that low angle refocusing pulses not fulfilling the Hahn spin echo conditions are primarily responsible for significant deterioration of T_1_-weighted spin echo image contrast in high-field MRI.

## 1. Introduction

Hahn spin echo (or simply SE) is the fundamental sequence of MRI with a 90° excitation and 180° refocusing pulses and offers specific tissue contrast depending on the T_1_, T_2_, and hydrogen atom density of the tissue. After a 90° excitation pulse rotates the tissue magnetization from the direction of main magnetic field (*z*) to the axial plane (*x* and *y*), there is little magnetization left along the main magnet, but the magnetization from axial plane begins to grow back within a fraction of a second for white and gray matter and somewhat slower for fluids. This rate of relaxation or signal loss (called T_1_ relaxation) depends on tissue chemistry and magnet strength and is the fundamental source of contrast in T_1_-weighted images [1]. If we were to create an image when these tissue times were widely separated, we would produce an image that has high contrast between these tissues, while if individual tissues are subjected to reduced or complex RF pulses, some of the tissue components may be discriminated and others may be preferentially detected leading to altered T_1_-weighted signal, and the image would not have true T_1_-weighted contrast. A faster version that maintains most of the SE advantages but with slight image blurring and a small contrast penalty is fast spin echo (FSE) in low to midfield magnets. 

Although we have primarily focused on the T_1_ effects that manifest through larger dimensions of the order of macromolecules or cells, there is another interaction (that drains detectable signal approximately 10 times faster than T_1_, called T_2_ relaxation) arising from physical and chemical couplings of atomic dimensions from neighboring spins (proton) which is sensitive to the magnetic impurities in the tissue rather than the magnet strength or cellular chemistry.

With the ever-increasing number of high-field MRI systems for diagnostic and research applications, there seems to be a loss in *in vivo* tissue contrasts [2]. This is of concern, particularly for T_1_-weighted imaging involving comparison with previous imaging results (often at lower fields) or for follow-up evaluations needing to replicate previous imaging contrast. This issue also exists in several T_2_-weighted imaging sequences but may be moderately alleviated as proposed by Weigel et al. [3] and other workers [4, 5]. The loss of T_2_-weighted image contrast may be attributed to magnetization transfer (MT) in multislice fast spin echo (FSE) sequence *in vivo* [3]. 

One may expect that by applying a slowly varying group of a large number of refocusing pulses with low angles the contrast for T_2_-weighted imaging may be preserved in spite of deviations from the Hahn spin echo condition and a significant presence of MT. Following this approach, there has been progress in designing 3D FSE sequences [5–9] that recover some of the lost T_2_ contrast for several tissues but cannot be easily translated to preserve T_1_-weighted contrast in high-field FSE sequences. 

In addition, tissue heating from radiofrequency pulses (measured by specific absorption rate or SAR) is an undesirable side effect in MRI [4] and increases quadratically with field strength. Since 180° refocusing pulses generate maximum heat, it is difficult to safely apply several of these pulses within short repetition time intervals at 3 T, as is needed in FSE T_1_-weighted imaging. There is a trend to use alternative T_1_-weighting methods that do not generate as much SAR; for example, one may use gradient echo imaging at high fields (MPRAGE or SPGR [5]) or use a typical high-field choice of 120° refocusing angle for 2D or 3D FSE with slow transition to pseudo steady states [6]. Unfortunately, a slowly varying long refocusing pulse train employs long TE and does not match the tissue contrast of low TE spin echo or FSE T_1_ sequences [7]. 

We wanted to investigate this situation with a simple, direct experiment as follows: applying a spin echo-based T_1_-weighted imaging that is fully compliant with the Hahn spin echo condition to macromolecule free tissue pairs with single and multislice options to test potential contrast loss as the refocusing angles are lowered, and a high field MR system is employed. The experiments were designed to evaluate intrinsic contrast preserved in T_1_-weighted spin echo sequences since for this group of sequences, the contrast loss, tissue heating, and image quality degradation at high fields for low-contrast objects seem to be a significant diagnostic and quality issue. 

## 2. Materials and Methods

### 2.1. Phantom Description and Imaging Slice Placement

**Figure 1:**
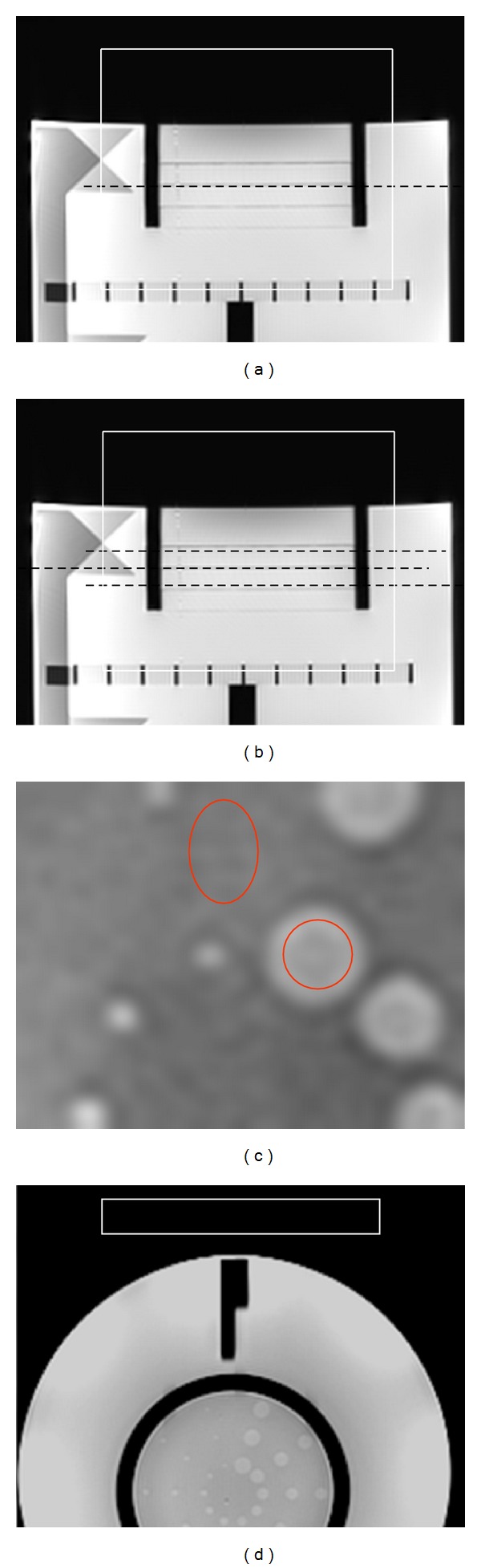
Image slice plan, shim volume, and ROI locations. (a) Image slice (dotted line) for single slice and (b) for 3-slice imaging plans. Also shown is the shimming volume used to ensure consistent field homogeneity during repeat experiments. (c) ROI, used: circular; spoke solution; oval; background solution: (d) rectangle; noise ROI.

The phantom is filled with 10 mmol nickel chloride (NiCl_2_) solution and 45 mmol sodium chloride to simulate biological conductivity and has been used for assessing clinical imagers [10]. Out of four low-density contrast disks located on the superior end of the phantom, the third one was placed at the isocenter with single slice imaging plan and the middle dotted line for the 3-slice plan. The image slice included an embedded thin sheet of polycarbonate plastic 0.006 inch or 0.15 mm in thickness. Partial volume contributions of the NiCl_2_ solution and the plastic sheet produce variations in signal strength (intrinsic contrast 3.6%) which may be used to visually assess the contrast performance of the sequence to distinguish low-contrast objects. Figures 1(a) and 1(b) show image slice positioning, shim volume used for achieving consistent field homogeneity and ROI measurement locations. In Figures 1(c) and 1(d), ROIs used are circular: for spoke solution; oval: for background solution containing plastic sheet; rectangular: for noise outside image area.

### 2.2. MRI Sequence and Parameters

Two T_1_-weighted sequences (spin echo and fast spin echo) were used at two different field strengths (1.5 T and 3 T), both GE HDx systems at 16.0 software platform and with transmit/receive head coils with identical designs. Typical scan parameters at 1.5T were TR = 200 ms/TE 12 ms/excitation flip angle 90°/refocusing flip angle = 180° or 120°/number of slices = 1 or 3/freq × phase matrix = 256 × 256/slice thickness/gaps = 5/5 mm/signal averages = 1/field of view = 25.6 cm/voxel resolution 1 × 1 × 5 mm^3^/scan time = 51 sec for single slice and 2 : 33 min for 3 slice spin echo acquisitions. With echo train length (ETL) of 3, the FSE T_1_ scan time was 17 sec for single slice and 51 sec for multi-slice acquisitions. Due to T_1_ lengthening at 3 T the TR chosen was 350 ms. All other parameters were kept the same as 1.5 T. 

### 2.3. Objective Measurement of Relative Contrast

**Figure 2:**
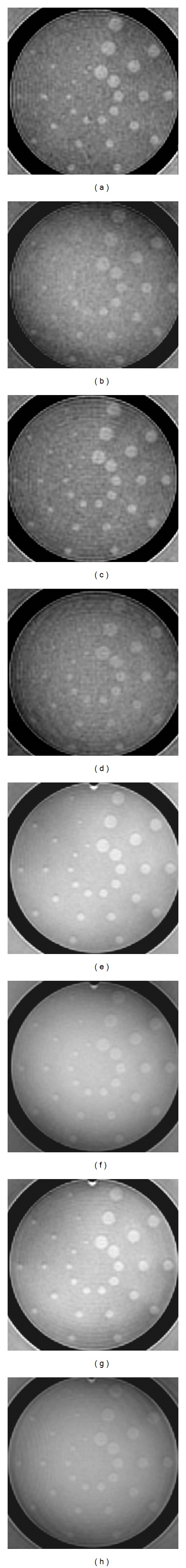
Spin echo (SE) and fast spin echo (FSE) images at 1.5 T (a)–(d) and at 3 T (e)–(h). (a) SE (CNR 3.8 for 180° refocusing), (b) SE (CNR 1.8 for 120° refocusing), (c) FSE (CNR 3.2 for 180° refocusing), (d) FSE (CNR 1.7 for 120° refocusing), (e) SE (CNR 3.8 for 180° refocusing), (f) SE (CNR 1.0 for 120° refocusing), (g) FSE (CNR 3.8 for 180° refocusing), and (h) FSE (CNR 1.1 for 120° refocusing); Note that 180° refocusing pulses produce 2–4 times the CNR present at 120° depending on the main magnetic field. Also, overall signal at 3 T is higher than that at 1.5 T.

**Figure 3:**
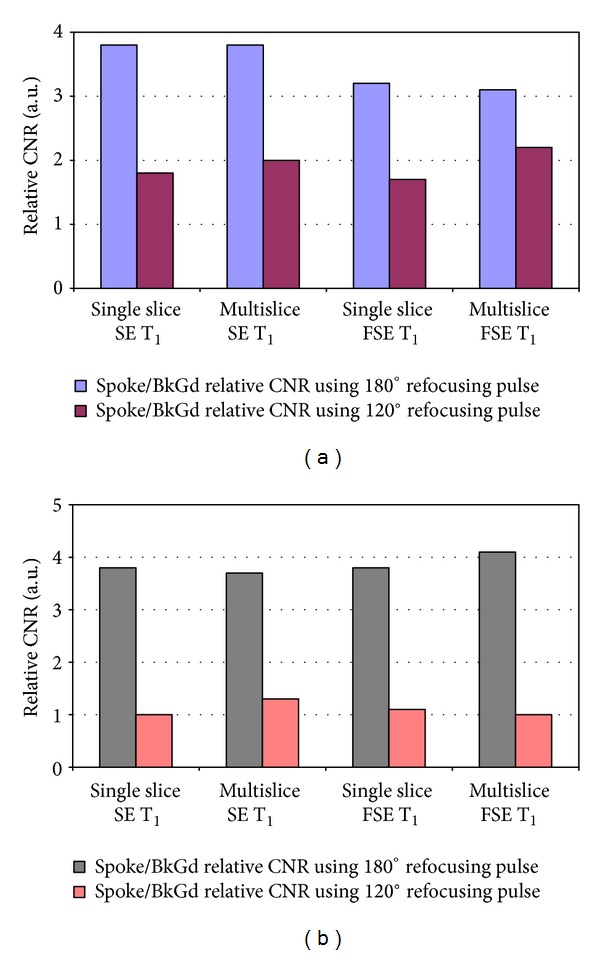
(a) Relative CNR at 1.5 T for spoke solution and plastic-embedded background solution using 180, and 120° refocusing pulses for SE and FSE with single or multislice imaging. (b) Relative CNR at 3 T for spoke solution and plastic-embedded background solution using 180 and 120° refocusing pulses for SE and FSE with single or multislice imaging.

The largest hole was chosen and background next to it for drawing low-contrast ROIs (circular and oval). Four rectangular noise ROIs were placed outside the phantom (only one shown, Figure 1(d)), and the average standard deviation was used for CNR computation. Percentage difference in CNR between the two low-contrast ROIs (circular and oval) was computed (Table 1) and plotted in Figures 2 and 3 for the two field strengths.

### 2.4. Theoretical Estimation of Relative Contrast

Spoke holes are 5 mm thick and allow full 5 mm thick image voxels containing only NiCl_2_ solution (circular ROI), while out of 5 mm background solution, 4.85 mm thickness was occupied by NiCl_2_ solution and 0.15 mm by the polycarbonate sheet. This led to a theoretical signal ratio of 5.00/4.85 = 1.032 or a relative contrast of 3.2% between the spoke and the background solution under proton density condition.

## 3. Results

### 3.1. Qualitative Assessments: Single and Multislice Results

Two readers independently assessed the images and agreed upon the following qualitative comparisons. The image appearance was somewhat mottled at 1.5 T. Multi-slice imaging produced higher signal compared to single slice. There were more Gibbs ringing artifacts noticeable for 120° refocusing pulses compared to images using 180° refocusing. In all images, all 10 spokes were identifiable in the image plane.

Figure 2 shows spin echo (panels (a), (b), (e), and (f)) and fast spin echo (panels (c), (d), (g), and (h)) images. Of these, the first group (panels (a)–(d)) is for 1.5 T and the second group ((e)–(h)) is for 3 T. The left-sided images ((a), (c) and (e), (g)) show higher contrast from 180° refocusing pulse, while right columns for the two groups ((b), (d) and (f), (h)) show reduced contrast due to 120° refocusing pulses.

### 3.2. Quantitative CNR Estimates

Two absolute contrast-to-noise ratios (CNR) estimated using the ROIs are grouped separately for the two field strengths in Table 1. Note that at 1.5 T in Table 1, the objective CNR for 180° refocusing pulses is almost twice of the CNR obtained from 120° pulses. The situation is worse at 3 T; the CNR is about 3-4 times for 180° refocusing pulses as compared to CNR using 120° pulse. 

The ROI-based objective CNR measurements were plotted in Figure 3 and compared for the two field strengths ((a), (b)) demonstrating significant CNR loss, particularly at 3 T when 120° refocusing pulses were used (a typical industry practice) for both SE and FSE T_1_ for both single and multi-slice imaging. There were no appreciable differences among single and multi-slice imaging-based contrasts for the 100% interslice gap chosen here.

## 4. Discussion

Three-echo-train FSE T_1_ in this experiment design produces as high a contrast as SE T_1_ but only at 3 T and not at 1.5 T. This may be due to a preferential signal loss only for the background solution at 3 T during the effective TE (TE = 12). Compared to bulk solution, the background solution might lose more signal due to bound spin layers on plastic surfaces that lower background signal more at 3 T than at 1.5 T since bound layers are known to have shorter T_2_. Although Constable et al. [11] and Melki et al. [12] have provided arguments toward basic contrast differences between conventional and fast spin echo sequences, the contrast loss for T_1_-weighted SE and FSE at higher fields has not been adequately explored and may require dedicated experiments similar to the ones proposed here and more. 

Toward understanding this issue, we draw attention to the significance of the loss of relative contrast in low refocusing situations at 3 T (use of 120° refocusing pulses for both spin echo and fast spin echo). Paschal and Morris [13] have suggested that central *K*-space imaged with large flip angles followed by *K*-space edges mapped with low refocusing angles should preserve MR contrast. This is the basis of 3D FSE imaging [14], but one may question whether long echo trains (with high to low switching for refocusing angles within a short TE period) can ever be a good solution for T_1_ spin echoes.

In our case, the loss is approximately 2/3 at 3 T for spin echo sequence requiring a single refocusing pulse and FSE with 3 refocusing pulses. It is also approximately 1/3 at 1.5 T which is still significant. One may argue that at 1.5 T, one may not usually need to use low refocusing pulses due to tolerable SAR involved at lower fields. However, the loss of 30% contrast with low refocusing angles even at 1.5 T is alarming and requires further experimentation. Note that in all our experiments, we employed a reproducible, first-order shimming routine and collected a full echo to minimize signal loss due to uncontrolled factors. 

However, this study was not designed to explore the effects present in all types of MRI sequences or to span the complete spectrum of low refocusing pulses with all possible interslice gaps. The choice of two widely used flip angles 180° and 120° represents a common industry practice [7]. Similarly, the 5 mm slices with 5 mm gaps remove the possibility of volume averaging or slice crosstalk [3] while fairly representing interleaved acquisition mode which is a clinically feasible option in MRI. In our chosen phantom model, the adjacent slices do not induce any significant contrast change. This is similar to the results for CSF and fat in the T_2_-related work by Weigel et al. [3] although our tissue pair corresponds to body tissues other than CSF or fat and the results point to inadequacy of T_1_-weighted imaging under low refocusing conditions. With no detectable differences between single and multislice imaging, we believe that for interleaved, spin echo-based T_1_ imaging, the low refocusing pulses may be somewhat immune to magnetization transfer effects when the system is free of imageable macromolecules. 

When we extrapolate our findings to clinical models, the presumed effects are twofold. Based on our institutional experience, the lack of T_1_-weighted tissue contrast at high fields is not correctible by simple adjustment of sequence parameters, and clinicians may increasingly depend upon the gadolinium-infused MRI contrast to widen the T_1_ contrast scale. Secondly, some institutions including ourselves have tried to lengthen the clinical T_1_ sequences with signal averaging or use lower resolution and increase slice gaps with the hope to gain back the lost tissue contrast from a higher SNR standpoint, none of which have resolved this problem. 

Obtaining consistent, intrinsically accurate tissue contrast with a spin echo T_1_ plays an important role in follow-up MRI examinations in several clinical situations, for example, in the management of multiple sclerosis or for grading progression of glioblastoma, for detecting heterotopic gray matter in epilepsy as well as in surgical resection plans for infiltrating musculoskeletal masses like sarcoma, to name a few [2, 4]. In summary, at high-field MR, systems the loss of spin echo or FSE T_1_ tissue contrast has not been well understood, and the role of slice crosstalk versus magnetization transfer effects has not been sorted out. 

Clinically, the exact amount of contrast loss depends on tissue composition. A tissue pair with higher or lower intrinsic contrast would be affected somewhat differently than observed here. However, the primary result in this study points to a clinically serious implication that the intrinsic tissue contrast may not be represented correctly when low refocusing pulses without satisfying the Hahn spin echo condition are used in T_1_-weighted MRI. This may be alleviated with a complex sequence design tailoring the refocusing pulses that may be difficult to achieve within a short TE. The current industry practice utilizes other preparations, for example, as in 3D MPRAGE or 3D FLASH without establishing a convincing clinical equivalence with spin echo T_1_ for most of the body tissues at high fields. 

## 5. Conclusions

To our knowledge, this is the first demonstration highlighting that the use of low angle refocusing pulses in T_1_-weighted spin echo MRI affects intrinsic tissue contrasts and can cause significant tissue contrast loss at high-field systems (e.g., at 3 T). Using macromolecule free tissue models and single and multi-slice imaging, we demonstrate that such a contrast loss cannot be explained by magnetization transfer. Although it is a common practice in high-field clinical MRI to manage RF heating by adopting low angle refocusing pulses, this work demonstrates a direct relationship between such deviations from the Hahn spin echo condition and undesirable loss of tissue contrasts affecting diagnostic quality.

## Figures and Tables

**Table 1:** ROI-based disc contrasts at 1.5 and 3 T for SE and FSE T_1_ sequences containing only NiCl_2_ solution relative to background containing both plastic sheet and NiCl_2_ solution.

	Single slice SE T_1_ (a.u.)	Multislice SE T_1_ (a.u.)	Single slice FSE T_1_ (a.u.)	Multislice FSE T_1_ (a.u.)
NiCl_2_ filled ACR phantom at 1.5 T				
Disc/BkGd relative CNR using 180° refocusing pulse	3.8	3.8	3.2	3.1
Disc/BkGd relative CNR using 120° refocusing pulse	1.8	2.0	1.7	2.2
NiCl_2_ filled ACR phantom at 3 T				
Disc/BkGd relative CNR using 180° refocusing pulse	3.8	3.7	3.8	4.1
Disc/BkGd relative CNR using 120° refocusing pulse	1.0	1.3	1.1	1.0
